# Impact of date stamping on patient safety measurement in patients undergoing CABG: Experience with the AHRQ Patient Safety Indicators

**DOI:** 10.1186/1472-6963-8-176

**Published:** 2008-08-13

**Authors:** Laurent G Glance, Yue Li, Turner M Osler, Dana B Mukamel, Andrew W Dick

**Affiliations:** 1 Department of Anesthesiology, University of Rochester School of Medicine and Dentistry, Rochester, New York, USA; 2 Department of Medicine, State University of New York at Buffalo, Buffalo, New York, USA; 3 Department of Surgery, University of Vermont Medical College, Burlington, Vermont, USA; 4 Department of Medicine, University of California, Center for Health Policy Research, Irvine, California, USA; 5 RAND Corporation, Pittsburgh, Pennsylvania, USA

## Abstract

**Background:**

The Agency for Healthcare Research and Quality (AHRQ) Patient Safety Indicators (PSIs) provide information on hospital risk-adjusted rates for potentially preventable adverse events. Although designed to work with routine administrative data, it is unknown whether the PSIs can accurately distinguish between complications and pre-existing conditions. The objective of this study is to examine whether the AHRQ PSIs accurately measure hospital complication rates, using the data with present-on-admission (POA) codes to distinguish between complications and pre-existing conditions

**Methods:**

Retrospective cohort study of patients undergoing isolated CABG surgery in California conducted using the 1998–2000 California State Inpatient Database. We calculated the positive predictive value of selected AHRQ PSIs using information from the POA as the gold standard, and the intra-class correlation coefficient to assess the level of agreement between the hospital risk-adjusted PSI rates with and without the information contained in the POA modifier.

**Results:**

The false positive error rate, defined as one minus the positive predictive value, was greater than or equal to 20% for four of the eight PSIs examined: decubitus ulcer, failure-to-rescue, postoperative physiologic and metabolic derangement, and postoperative pulmonary embolism or deep venous thrombosis. Pairwise comparison of the hospital risk-adjusted PSI rates, with and without POA information, demonstrated almost perfect agreement for five of the eight PSI's. For decubitus ulcer, failure-to-rescue, and postoperative pulmonary embolism or DVT, the intraclass-correlation coefficient ranged between 0.63 to 0.79.

**Conclusion:**

For some of the AHRQ Patient Safety Indicators, there are significant differences in the risk-adjusted rates of adverse events depending on whether the POA indicator is used to distinguish between pre-existing conditions and complications. The use of the POA indicator will increase the accuracy of the AHRQ PSIs as measures of adverse outcomes.

## Background

The Institute of Medicine (IOM) report *To Err is Human *[1] has fundamentally changed the landscape of American medicine by challenging us to examine and improve the safety and quality of the health care system in this country [2]. Seven years after the release of this seminal report, "the quality chasm in health care remains wide [3]." A recent study from RAND documented that adults in the United States receive only 55% of recommended care [4]. In its blueprint for "Crossing the Quality Chasm", the IOM recommended the development of performance and outcome measurements [5]. In response, the Agency for Healthcare Research and Quality (AHRQ) has constructed Patient Safety Indicators (PSI) [6] to serve as the "state-of-the art in measuring the safety of hospital care through the analysis of inpatient discharge data [6]."

The AHRQ PSIs provide information on risk-adjusted rates for potentially preventable adverse events such as decubitus ulcers, postoperative hemorrhage, and postoperative sepsis. In theory, a hospital can benchmark its performance using these safety indicators and target specific areas of poor performance for improvement. This 'global' approach to quality improvement is more likely to be effective than the traditional approach of narrowly focusing on the unsafe actions of a few physicians [7]. Traditional quality assurance (QA) efforts, which are triggered by individual case reviews, may divert limited hospital resources to clinical areas where overall hospital performance is not problematic, while ignoring system-level problems. Using PSIs to guide QI efforts may force hospitals to focus their efforts on the development of systems which will reduce the incidence and mitigate the impact of dangerous medical errors [7].

The Patient Safety Indicators were designed to be used with administrative data and are currently being used by public and non-public organizations to assess hospital quality of care [8]. Basing the PSIs on administrative data makes it possible for virtually any hospital to examine its safety performance. Hospitals can download the PSI software from the AHRQ web site and use their own administrative data to construct risk-adjusted PSI rates and compare themselves to a national benchmark. Because these indicators are based on administrative data, there is no added data collection burden. However, administrative data have well recognized limitations, including the lack of precise definitions and coding differences across hospital [9]. Several investigators have questioned the accuracy of using administrative data to identify complications [10,11], while others have concluded they may be useful for identifying surgical complications [12].

One of the primary problems with using administrative data to identify adverse events is the failure of most ICD-9-CM codes to distinguish between pre-existing conditions and complications that occur after hospital admission. Counting pre-existing conditions as adverse events may falsely elevate adverse event rates and bias these safety indicators against hospitals which care for sicker patients. In designing the PSIs, AHRQ researchers attempted to exclude ICD-9-CM codes that could represent either a pre-existing condition or a complication [13]. In theory, the use of a present-on-admission (POA) modifier would have made it possible to avoid mis-classifying pre-existing conditions as adverse events. Several states (California, New York State, Florida, and Wisconsin) require hospitals to include a POA modifier in their administrative data. However, AHRQ researchers were initially constrained to develop a set of indicators which could be used by hospitals in all States, and thus were not able to incorporate the POA indicator in the PSI algorithms [6]. The latest version (2007) of the AHRQ PSIs incorporates the POA indicator and gives users the options of using this information to calculate the AHRQ PSIs [14].

Using the POA modifier to differentiate pre-existing conditions from complications, we will examine the accuracy of the AHRQ PSIs using discharge abstracts for patients undergoing coronary artery bypass graft (CABG) surgery in the California State Inpatient Database. Previous studies have shown that the AHRQ PSIs over-estimate the number of patient safety events when pre-existing conditions are incorrectly counted as complications [15,16]. One of these studies, by Houchens and colleagues [16], specifically examined the impact of counting pre-existing conditions as complications on hospital PSI rates based on all patient admissions. However, both studies are based on a broad range of patient diagnoses and procedures. Although it is likely that there is some overlap in the care processes that prevent certain complications across different surgical procedures and medical conditions, it is also likely that specific best practices for preventing complications may be unique to different patient populations. For example, it is unlikely that one hospital-wide approach to preventing postoperative hemorrhage will be equally effective in patients undergoing hysterectomy compared to CABG patients, because the causes of postoperative hemorrhage in these two groups are so different. Just as procedure-specific mortality rates (e.g. CABG) are more informative, and possibly more useful, than overall hospital mortality rates, we believe that diagnosis-specific PSI rates may prove more useful that hospital-wide PSI rates.

We have previously shown that the addition of date stamp information to administrative data has a substantial impact on the ranking of hospital quality based on risk-adjusted mortality rates [17,18]. In the current study, our goal is to examine whether the AHRQ PSIs accurately measure the rates of adverse events, using the data with the POA modifier as the gold standard for comparison. Under the Deficit Reduction Act of 2005, the Centers for Medicare and Medicaid Services will require hospitals to add POA indicators to Medicare claims starting in 2007 [19]. The results from this study will help to inform policy-makers as to the value of requiring the inclusion of the POA modifier in non-Medicare claims as well.

## Methods

### Data

This analysis of patients undergoing isolated CABG surgery was conducted using the 1998–2000 California State Inpatient Database, which contains 100 percent of the state's inpatient discharge records. The data were obtained from the Healthcare Cost and Utilization Project (HCUP). Each patient record has ICD-9-CM coding slots for up to 30 diagnoses. Except for E-codes, each ICD-9-CM code is modified by a POA code that specifies whether a diagnosis was present at the time of hospital admission. Although not fully validated, previous work suggests that the POA field has clinical validity [17,19-23].

The CABG study population was identified using ICD-9-CM codes 36.10 – 36.19 and 36.2. We excluded 557 patients with missing hospital identifiers, age less than 18 years, missing gender, or missing discharge status. In order to eliminate hospitals which may be coding the POA modifier inaccurately, we excluded 13 hospitals (n = 2593 patient discharges) that coded POA = yes or POA = no for every record, hospitals for which greater than 10% of the POA codes were missing, and hospitals whose percent of ICD-9-CM codes coded as present-on-admission was outside of the 95% confidence interval for the CABG patients in this data set (0.67, 0.91). The final patient population consisted of 82,063 patients from 111 hospitals.

### Identification of adverse events

The AHRQ Patient Safety Indicator (PSI) software [6] was used to calculate event rates for adverse events. These indicators were developed by the AHRQ Evidence-Based Practice Center at the University of California and Stanford [6]. The selection and grouping of ICD-9-CM codes into PSIs was based on the Complications Screening Program [24], developed by Iezzonni, and a comprehensive review of existing ICD-9-CM codes [6]. The validity of these indicators was evaluated using the RAND/UCLA Appropriateness method [25] and empirical analyses to assess the frequency, variance, and bias of these indicators [6]. Risk adjustment is performed by the PSI software using logistic regression models which are based on the 29 states in the 2000 HCUP State Inpatient Databases. These models adjust for differences in casemix as defined by age, sex, modified DRGs, and the AHRQ Elixhauser comorbidity diagnostic categories [26,27].

We selected eight PSIs which had relatively high event rates and were clinically relevant for patients undergoing CABG surgery:

#### Decubitus Ulcer

Patient records with secondary diagnosis of decubitus ulcer and length-of-stay greater than 4 days. Patient exclusions include any diagnosis of hemiplegia, paraplegia, or quadriplegia; and patients admitted from a long-term facility or transferred from an acute care facility [6].

#### Failure-to-Rescue

Patient records in which the discharge disposition is death and which indicate a potential complication of care during the hospitalization (i.e., pneumonia, DVT/PE, sepsis, acute renal failure, shock/cardiac arrest, or GI hemorrhage). Patient exclusions include age greater than 75 years; and patients admitted from a long-term facility or transferred from an acute care facility [6].

#### Infection due to Medical Care

Patient records with secondary diagnosis of infectious complication of medical care; or infection due to other vascular device, implant or graft. Patient exclusions include length-of-stay less than 2 days, any diagnosis code for immunocompromised state or cancer, or cancer DRG [6].

#### Postoperative Hemorrhage or Hematoma

Records of patients with secondary diagnosis of postoperative hemorrhage or hematoma who required postoperative control of bleeding or a drainage procedure. Cases were excluded if a procedure for postoperative control of bleeding or a drainage procedure was the only procedure in the record, or occurred prior to the operative procedure [6].

#### Postoperative Physiologic and Metabolic Derangement

Patient records with secondary diagnosis of physiologic and metabolic derangements. Cases were excluded it the records included ICD-9-CM codes for chronic renal failure; acute renal failure where dialysis occurs prior to or on the same day as the operative procedure; or a primary diagnosis of diabetes and a secondary diagnosis code for ketoacidosis, hyperosmolarity, or coma [6].

#### Postoperative Pulmonary Embolism (PE) or Deep Venous Thrombosis (DVT)

Patient records with secondary diagnosis of DVT or PE. Cases were excluded if a procedure for interrupting the vena cava is (1) the only operative procedure or (2) occurs before or on the same day as the operative procedure [6].

#### Postoperative Sepsis

Patient records with secondary diagnosis of sepsis. Cases were excluded if the principal diagnosis was infection, if any of the diagnoses included immunocompromised state or cancer, or if the length-of-stay was less than 4 days [6].

#### Accidental Puncture of Laceration

Patient records with secondary diagnosis of accidental cut, puncture, perforation, or laceration [6].

### Impact of POA indicator

In order to analyze the impact of the POA indicator on the PSIs, we modified the AHRQ PSI algorithm to make use of the information from the POA indicator to exclude ICD9 codes that were present-on-admission and thus could not be complications. With the exception of the Failure-to-Rescue PSI, only the numerator for the PSIs was modified. For failure-to-rescue, only the denominator was modified since the numerator equals the number of deaths, whereas the denominator represents complications (only cases where the POA indicator indicated that the condition was not present on admission were included in the denominator for the modified PSIs). We also modified the AHRQ Elixhauser comorbidity algorithm by only including secondary diagnoses present-on-admission for risk adjustment. We then compared the PSI obtained using the "modified" PSI algorithms versus the "standard" PSI algorithm which ignored information contained in the POA modifier. This analysis was performed first at the level of individual patients, and then at the hospital-level. For the hospital level analysis, we calculated the intra-class correlation coefficient to assess the level of agreement between the point estimates of the risk-adjusted PSI rates obtained with and without the information contained in the POA indicator [28].

The AHRQ PSI algorithms were run using SAS version 8.2 (SAS Corp., Cary, NC). This study was exempted from review by the University Of Rochester School Of Medicine Research Subjects Review Board.

## Results

Overall, the false positive error rate for the PSIs, defined as one minus the positive predictive value, ranged between 7% for "infection due to medical care" to 41% for "decubitus ulcer" (Table 1). The false positive error rate was greater than or equal to 20% for four of the eight PSIs: decubitus ulcer, failure-to-rescue, postoperative physiologic and metabolic derangement, and postoperative pulmonary embolism or DVT. The observed rates per 1000 discharges at risk differed significantly (P-value ≤ 0.05) for six of the eight PSIs, and was marginally significant for one of the eight (P-value = 0.06).

**Table 1:** Impact of the POA indicator on observed rates of adverse events.

Patient Safety Indicators	No. of Events		Risk Pool		1-PPV	Observed Rates per 1000 Discharges at Risk	
			
	No POA	POA	No POA	POA		No POA	POA
Decubitus Ulcer	228	135	49,463	49,463	0.41	4.61†	2.73†
Failure to Rescue	403	324	3298	2,095	0.20	122.2†	154.7†
Infection due to Medical Care	374	349	72,954	72,954	0.07	5.13	4.78
Postop Hemorrhage or Hematoma	524	455	82,046	82,046	0.13	6.39†	5.55†
Postop Physiologic & Metabolic Derangement	191	153	35,003	35,003	0.20	5.46†	4.37†
Postop Embolism or DVT	512	343	82,040	82,040	0.33	6.24†	4.18†
Postop Sepsis	247	207	30,054	30,054	0.16	8.22¥	6.89¥
Accidental Puncture or Laceration	961	848	82,050	82,050	0.12	11.71†	10.34†

† P-value ≤ 0.05

¥ P-value = 0.06

DVT – deep venous thrombosis, POA – present-on-admission indicator; PPV – positive predictive value; obs – observed; postop – postoperative

**Figure 1:**
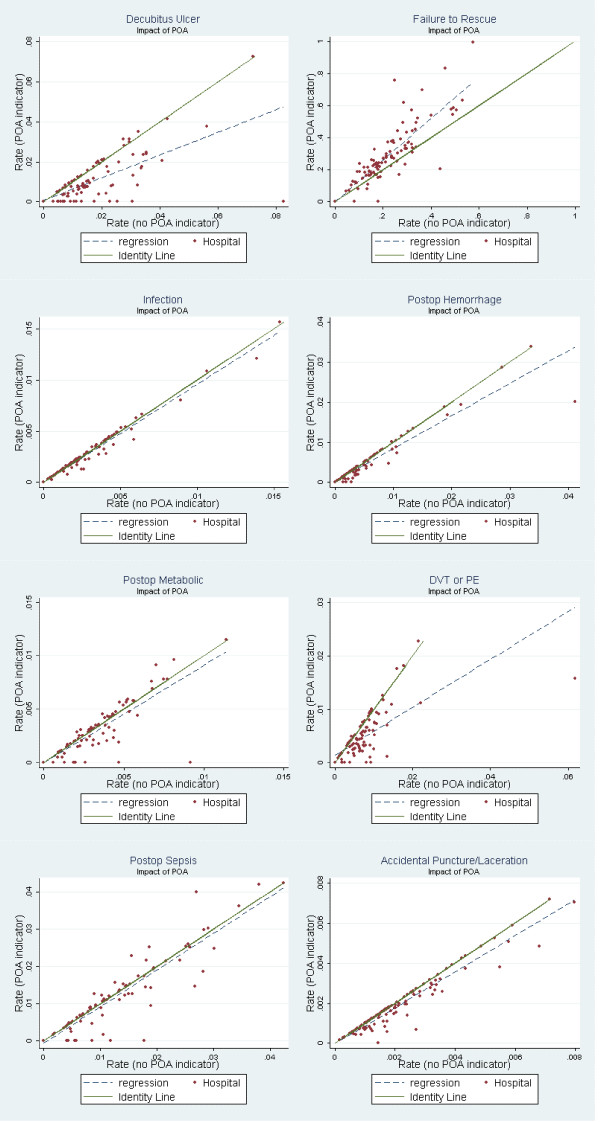
**Comparison of hospital risk-adjusted PSI rates based on the *standard *PSI algorithm versus *modified *PSI algorithm**. *standard PSI algorithm *– does not use information from the POA field to distinguish pre-existing conditions from complications *modified PSI algorithm *uses information from the POA field to distinguish between pre-existing conditions and complications identity line is a 45-degree line which corresponds to perfect agreement for risk-adjusted PSI rates based on the 'standard' and 'modified' PSI algorithms.

The comparisons of the hospital risk-adjusted PSI rates obtained using the *standard algorithm *(does not use the POA field to distinguish pre-existing conditions from complications) and the *modified algorithm *(uses the POA field to distinguish between pre-existing conditions and complications) is shown in Figure 1. Pairwise comparison of these risk-adjusted PSI rates, with and without POA information, demonstrated almost perfect agreement for five of the eight PSI's (intraclass correlation coefficient: 0.81–1.00). In the case of "decubitus ulcer", "failure-to-rescue", and "postoperative pulmonary embolism or DVT", there was significant deviation of the regression line (standard versus modified risk-adjusted PSI rate) from the 45-degree line, and the intraclass-correlation coefficient ranged between 0.63 to 0.79 (Table 2 and Figure). With the exception of failure-to-rescue, adding the POA indicator generally decreases a hospital's PSI rates because the original PSI algorithm is flagging conditions as complications that were pre-existing conditions. In the case of failure-to-rescue (FTR), adding POA information lowers the PSI rate because the risk pool for FTR is made up of patients with complications, and fewer patients are flagged as having complications using the POA indicator.

**Table 2:** Extent of agreement of hospital risk-adjusted PSI rates based on 'standard' versus 'modified' PSI algorithm.

Patient Safety Indicators	Intraclass Correlation Coefficient
Decubitus Ulcer	0.666
Failure to Rescue	0.791
Infection due to Medical Care	0.988
Postoperative Hemorrhage or Hematoma	0.935
Postoperative Physiologic & Metabolic Derangement	0.871
Postoperative Embolism or DVT	0.628
Postoperative Sepsis	0.931
Accidental Puncture or Laceration	0.954

DVT – deep venous thrombosis

## Discussion

In this study we find that the present-on-admission (POA) indicator has a significant impact on some of the AHRQ Patient Safety Indicators (PSI) rates in patients undergoing CABG surgery. The PSIs are one component of the quality toolbox developed by AHRQ to facilitate quality improvement and provide hospitals with the opportunity to benchmark their performance [29,30].

In practice, quality assurance is usually triggered by case reviews and focuses on the perceived failures of individual physicians and providers. Medical errors are attributed to "aberrant mental processes such as forgetfulness, inattention, poor motivation, carelessness, negligence, and recklessness [7,31]." Since individual cases selected for examination are often reviewed in isolation, as opposed to being reviewed as part of a cohort of similar cases, the critical role of health care systems in causing medical errors is frequently ignored. Because PSI rates are, by construction, a measure of global hospital performance, they shift the focus of error analysis from the individual provider to the level of the health care system. For example, a high rate of postoperative sepsis after CABG surgery across cardiac surgeons is more likely to improve with better adherence to patient safety practices, such as the use of maximum sterile barriers during catheter insertion or the use of antibiotic-impregnated catheters [32], than by the act of "disciplining" a single physician. Thus, PSIs may provide the impetus for a hospital's leadership to examine the "latent conditions" that lead to medical errors – production pressure, inadequate staffing, fatigue – and help set the stage for the adoption of a true "systems approach" to reducing medical error and improving health care quality.

The AHRQ PSI rates have the advantage of being based on administrative data, which are collected by virtually all hospitals in computerized form, and thus is readily available at low cost. Furthermore, the availability of the AHRQ PSI software in the public domain provides all hospitals with the opportunity to benchmark and track their PSI rates. However, the use of administrative data to monitor complications also has important limitations. In particular, the under-reporting of complications using ICD-9-CM codes, in addition to variability in coding practices across institutions, raises questions regarding the validity of using ICD-9-CM codes to report complications [33] and creates concerns that public reporting of PSI rates may unfairly penalize those hospitals with more accurate reporting practices. The primary limitation of this study is the assumption that the POA indicator accurately distinguishes complications from pre-existing conditions. Parker and colleagues [23] recently examined the accuracy of administrative data from California, using the POA indicator to exclude complications, with a clinical registry for CABG patients. Using the clinical data as the gold standard, the sensitivity of the risk factors in the administrative data ranged between 22% to 95%, with most above 50%. For most risk factors, specificity exceeded 90%. AHRQ has recently released a report summarizing the evidence supporting the value and validity of the POA indicator [19].

However, these well recognized limitations of administrative data for error reporting should not prevent individual hospitals or hospital systems from using non-public reports based on the AHRQ PSI to facilitate quality improvement. Despite the inherent limitations of risk-adjustment for "leveling the playing field" [34,35], public and non-public reporting of hospital mortality rates have been associated with significant decreases in mortality for cardiac [36,37] and non-cardiac surgery [38].

Our findings in this study examine one of the known limitations of administrative data for error reporting, namely, the inability of administrative data to effectively distinguish between pre-existing conditions and complications. Despite the fact that the AHRQ PSI were designed to "emphasize specificity over sensitivity", we found significant numbers of false positives for some of the PSIs. The planned expansion of the use of the POA indicator to all Medicare claims, beginning in 2007, could improve the validity of the AHRQ PSIs if the AHRQ algorithm were revised to include the POA indicator. Recent research in the private sector has led to the development of a system to identify Potentially Preventable Complications (PPC) [21]. By incorporating the POA indicator into its algorithms for the PPC groups, it was possible to expand the number of diagnoses that could be considered complications without sacrificing specificity. This expansion in scope of error monitoring, predicated on the use of the POA indicator to distinguish complications from pre-existing conditions, may be the "next step" in the evolution of the AHRQ PSI. This "next-generation" complication reporting system may provide greater opportunities for reducing medical errors and improving health care quality. Our study, by showing significant number of false positives using the AHRQ PSIs, further reinforces the need for the widespread adoption of the POA indicator which will make it possible for revised PSI systems, such as the PPC system, to be widely adopted.

Two recent studies have investigated the impact of the POA indicator on patient safety events. The first, by Naessens and colleagues [15], was based on hospital discharges from the Mayo Clinic Rochester hospitals. This study found that after eliminating secondary diagnoses that were present on admission, the overall rate of patient safety events decreased by nearly 50%. The second, by Houchens and colleagues [16], used data from California and New York State Inpatient Databases to examine the impact of the POA indicator. This study found that three of the 13 PSIs greatly over-estimated the number of patient safety events when information from the POA indicator was not used to differentiate pre-existing conditions from complications. For these three PSIs, there were significant discrepancies between hospital risk-adjusted PSI rates before and after excluding pre-existing conditions. Our study adds to the existing literature by focusing on a single medical condition, CABG surgery, as opposed to basing the analysis on all inpatient admissions. We believe that PSIs will be useful only insofar as they allow physicians and hospitals to identify problems and, then to focus QI efforts for specific hospital departments, as opposed to solely providing hospitals with a global measure of patient safety events. In this light, studies evaluating the validity of the AHRQ PSIs should assess disease-specific performance, in addition to global performance. In addition, our previous work has shown that the extent to which complications are mis-identified as pre-existing conditions varies substantially across patient populations (e.g. CABG, abdominal aortic aneurysm repairs, stroke patients) [22]. Thus, it is likely that the accuracy of PSIs would also vary across patient groups. The accuracy of the AHRQ PSI in CABG patients may be of particular interest to hospitals seeking to improve CABG outcomes that only have access to administrative data without the POA indicator.

Increasingly, private payers and Medicare are promoting the use of financial incentives to improve the quality of care through pay for performance initiatives. Nationally, over fifty-percent of Health Maintenance Organizations covering greater than 80% of enrolled patients, have pay-for-performance programs in place [39]. Under the Deficit Reduction Act of 2005, the reduction in hospital Medicare payments to hospitals not reporting quality data will increase five-fold from 0.4 percent to 2 percent, and infectious complications will no longer entitle hospitals to higher reimbursement rates [40]. However, the actual impact of pay-for-performance on quality is largely unknown [41], although recent work suggests that financial incentives has a relatively modest effect on adherence to process measures [42]. Even if financial incentives were found to significantly improve adherence to process measures, recent work suggests that adherence to "best practices" has only a relatively modest impact on risk-adjusted 30-day mortality rates for patients with acute myocardial infarctions (0.6%), heart failure (0.1%) and pneumonia (0.1%) [43]. In light of the weak association between many processes of care and outcome, direct outcome measures, such as PSI and related measures of adverse events, may have an important role in future efforts to improve health care quality.

## Conclusion

For some of the Patient Safety Indicators, there are significant differences in the rates of adverse events depending on whether the POA indicator is used to distinguish between pre-existing conditions and complications. The use of the POA indicator will increase the accuracy of the AHRQ PSIs as measures of adverse outcomes, and will make the future implementation of more comprehensive measures of complications more feasible.

## Competing interests

The authors declare that they have no competing interests.

## Authors' contributions

LGG conceived and designed the study, performed the statistical analysis, and drafted the manuscript. YL modified the computer algorithm used to calculate the AHRQ PSIs and contributed to the drafting of the manuscript. TMO, DBM, and AWD participated in the design of the study and contributed to the drafting and revision of the manuscript.

## Pre-publication history

The pre-publication history for this paper can be accessed here:


